# Challenging Popular Belief, Mosquito Larvae Breathe Underwater

**DOI:** 10.3390/insects15020099

**Published:** 2024-02-01

**Authors:** Agustin Alvarez-Costa, Maria Soledad Leonardi, Silvère Giraud, Pablo E. Schilman, Claudio R. Lazzari

**Affiliations:** 1 Institut de Recherche sur la Biologie de l’Insecte, UMR7261 CNRS—University of Tours, 37200 Tours, France; agustinalvarezcosta@gmail.com (A.A.-C.); leonardi@cenpat-conicet.gob.ar (M.S.L.); silvere.giraud@ird.fr (S.G.); 2 Instituto de Biodiversidad y Biología Experimental y Aplicada, IBBEA (CONICET-UBA), DBBE, University of Buenos Aires, Buenos Aires 1428, Argentina; schilman@bg.fcen.uba.ar; 3 Instituto de Biología de Organismos Marinos, IBIOMAR-CONICET, Puerto Madryn 9120, Argentina

**Keywords:** *Aedes*, metabolism, survival, respiration, temperature, Q_10_

## Abstract

**Simple Summary:**

We present the first simultaneous quantitative analysis of mosquito larval respiration in air and water, revealing the unknown ability of the larvae of the most cosmopolitan (*Aedes aegypti*) and the most invasive (*Aedes albopictus*) disease vectors to breathe underwater.

**Abstract:**

Immature mosquitoes are thought to breathe only atmospheric air through their siphons despite reports of prolonged submerged survival. We studied the survival of last-instar larvae of *Aedes aegypti* fully submerged at different temperatures and measured the oxygen consumption from air and water-dissolved larvae and pupae of this species under different conditions. Larvae survived much longer than expected, reaching 50% mortality only after 58, 10, and 5 days at 15°, 25°, and 35 °C, respectively. Larval to pupa molt was only observed in larvae with access to air, whereas individuals kept submerged never molted. Although most of the oxygen was obtained from the air, larvae obtained 12.72% of their oxygen from the water, while pupae took only 5.32%. In both media, temperature affected the respiration rate of the larvae, with relatively close Q_10_ values (1.56 and 1.83 for water and air, respectively). A similar pattern of O_2_ consumption was observed in *Ae. albopictus*, whose larvae obtained 12.14% of their oxygen from the water. The detailed quantification of oxygen consumption by mosquito larvae showed that water-dissolved oxygen is not negligible and physiologically relevant, challenging the idea that mosquito larvae only breathe atmospheric oxygen.

## 1. Introduction

As an originally terrestrial group and from an evolutionary point of view, when insects colonized fresh water, they had to deal with many vital functional problems. Respiration was probably the biggest. As an environment, water is a less favorable medium for respiration because its oxygen concentration is twenty times lower, and its oxygen diffusion rate is lower by a factor of 105 [1]. In this sense, insects adapted to aquatic environments have developed many different strategies to acquire oxygen. Examples include cutaneous respiration, the development of spiracular, tracheal, and blood gills, the use of air reservoirs with air bubbles, or the use of hydrofuges structures to remain attached to the water surface and breathe atmospheric air [2]. The latter is thought to be the case for mosquito larvae and pupae, which use their siphons as snorkels when resting suspended from the water surface. This statement is widely affirmed in textbooks, on scientific and educational websites, and in protocols for the control of mosquito populations at the juvenile stage. However, the vital role of aerial respiration in immature mosquitoes has been questioned by sporadic observations (e.g., [3,4,5,6,7]).

One of the first scientists interested in the possibility of aquatic respiration in mosquito larvae was the Brazilian entomologist Ângelo Moreira Da Costa Lima. More than a century ago, Da Costa Lima carried out a series of experiments in which larvae of various Culicidae species were kept completely submerged, and the status of each individual was reported day by day [3]. The author observed that some larvae survived for several days and that a larva whose “leaflets” (i.e., anal papillae) had been removed returned to the surface more often than another intact larva used as a control. These results led the author to state: “*The results of my experiments convinced me that mosquito larvae, while generally breathing mainly free air by the two tracheae of the respiratory siphon, also respire the oxygen of the air dissolved in water, the gaseous exchanges being made by the branchial leaflets and the general integument of the body.*”. This assertion was criticized by colleagues who distrusted the results due to the poor control of the experimental conditions [8]. Da Costa Lima [9] replicated some of the original experiments with additional care and reported similar observations to his earlier work. Da Costa Lima also noted the similarity of anal papillae to gills, in agreement with other colleagues who considered these structures to be respiratory organs in aquatic Diptera [10]. The demonstration of the osmoregulatory function of the papillae [11,12,13], together with the general assumption that the potential contribution of dissolved oxygen should be insignificant, quickly led to aquatic respiration being disregarded as physiologically relevant for mosquito larvae [14].

Although it seems reasonable to assume that atmospheric air is the main source of oxygen, it cannot be excluded that mosquito larvae and pupae may also obtain some oxygen from the water. On the one hand, underwater survival of larvae of different mosquito species and some measurements of oxygen consumption from the water [15,16] provided evidence that oxygen dissolved in water could maintain vital functions during periods without contact with the surface. However, the relative contribution of atmospheric and water-dissolved oxygen to the metabolism of mosquito larvae and pupae has not been exhaustively investigated.

The aim of this study is to provide further insights into the respiratory gas exchange in immature mosquito stages with atmospheric air and water. Two species, *Aedes aegypti* and *Ae. albopictus,* were chosen as experimental models given their relevance as vectors of numerous pathogens and their highly invasive potential. We analyzed the survival and oxygen uptake of mosquito larvae and pupae with and without access to air and measured the effect of temperature on these variables. For the first time, we provide quantitative data on oxygen consumption from air and water, measuring both separately and simultaneously. Finally, we discuss the findings of our study in the context of mosquito respiratory physiology.

## 2. Materials and Methods

### 2.1. Mosquitoes

Eggs of *Aedes aegypti* of the Bora strain (insecticide susceptible) and *Ae. albopictus* of Vectopole strain reared at Vectopole (Montpellier, France) were provided by the European network InFravec2 (https://infravec2.eu/). Eggs were put in dechlorinated tap water for hatching, adding traces of ascorbic acid and tropical fish food, and kept at 26 °C (±1 °C) in a climatic chamber, under a light/dark cycle 12 h:12 h (lights on at 08:00 am). Food was regularly provided until they reached the 4th instar (6–10 days in our conditions) and used for experiments. Individuals were handled by aspirating them with plastic Pasteur pipettes with cut tips. In all the experimental series, each larva or pupa was tested only once and discarded afterward.

### 2.2. Survival Experiments

The survival time of fourth-instar larvae of *Ae. aegypti* was evaluated at three different temperatures. For each temperature, two larvae were placed in containers with 300 mL of dechlorinated water under two conditions: one submerged and one with access to air. In both cases, a unique larva was placed into a glass cylinder (0.6 cm in diameter and 2 cm in length), both ends of which were closed by a tissue mesh held in place by an O-ring; this allowed water circulation, and at the same time kept the larva caged. In the first condition (submerged), the cylinder was completely sunk at the bottom of the container, taking care that no air remained captive inside. The control condition consisted of a larva placed in a similar vertical cylinder, which maintained the superior half in contact with the air and the inferior half underwater (see Figure S1). No food was provided, but the water was neither changed nor the development of microorganisms impeded. Containers were placed inside climatic chambers set at 15°, 25°, or 35 °C (±0.5 °C) under a 12h:12h light/dark cycle (lights on at 08:00 am). Relative humidity was set at 70 in order to minimize water evaporation in the containers. In case some evaporation occurred, dechlorinated water was added to return to the initial volume. The number of dead and molted insects was recorded daily. Ten containers were used simultaneously at 25 °C, and six containers were used at 15 °C and 35 °C. As the number of containers was limited, the larva that died or pupated during the experiment was replaced by a live one. The experiment ended when all the larvae placed in the container at the beginning of the experiment died or pupated except for 25 °C, where the experiment ended on day 29 because only one initial larva from the submerged treatment was still alive, and no mortality was observed in the control group.

### 2.3. Oxygen Consumption

The individual oxygen consumption of immature *Ae. aegypti* was measured using optodes. We employed two 4-channel Firesting O_2_ meters (Pyro Science, Aachen, Germany) using 4 mL vials with an integrated optical oxygen transducer (OXVIAL 4). Briefly, flashes of light of specific wavelengths generated in the interface are guided through a light fiber to excite a transducer inside the vial from the outside. The fluorescent of the substance, which is proportional to the oxygen concentration in the medium (air or water), is gathered by the same optic guide and analyzed at the interface. The temperature of the vials was controlled using a Peltier element and controller (QuickCool 34W; Peltron GmbH, Fürth, Germany). A temperature sensor from the oxygen meter measured the temperature inside an empty vial, and its signal allowed the system to adjust the values of the measured O_2_ concentrations.

Oxygen consumption of fourth-instar larvae of *Ae. aegypti* and *Ae. albopictus*, and pupae of *Ae. aegypti* were measured. The oxygen consumption of *Ae. aegypti* immature stages was measured under three vial conditions: submerged, closed vial, and open vial, at 25 °C. In addition, oxygen consumption was measured for the submerged condition (O_2_ obtained from water) and closed vial condition (O_2_ obtained from air) at 15 °C and 35 °C, respectively. All types of measurements (O_2_ obtained from water or air), different vial conditions (submerged, closed, and open vial), mosquito stage and species, as well as temperature of measurements, are summarized in the Supplementary Tables S1 and S2. The submerged condition involved placing an individual inside a closed vial filled with water, and the concentration of oxygen in the water was recorded for a duration of four hours. During this treatment, meticulous attention was given to ensuring the absence of any air bubbles within the vial. For the closed vial condition, a mosquito in its immature stage was introduced into a sealed vial that was half-filled with water. The levels of both water and air oxygen concentrations were measured over a span of four hours. In the open vial condition, an individual mosquito was placed inside a vial that was half-filled with water and left open. The concentration of oxygen in the water was recorded over a period of four hours. By comparing the results of these two experiments, we aimed to evaluate the diffusion or exchange of oxygen between the water and the air within the vial. Dechlorinated water was utilized in all assays, and each treatment was replicated between 12 and 30 times. To determine the rate of oxygen consumption for each replicate, we calculated the slope of a linear regression representing the O_2_ concentration over the duration of the experiments. For *Ae. albopictus*, similar assays were conducted, except for the open vial condition and the closed vial condition at 15° and 35 °C. All the measurements were carried out during daylight hours, from 10 a.m. to 6 p.m. For a complete list of measurement types, see Supplementary Tables S1 and S2. Vials were filled with water, but no larvae were used to determine the baseline of our measures.

### 2.4. Statistics

To analyze differences in survival across temperatures and conditions (submerged and controlled), a Kaplan–Meier analysis was performed. The Kaplan–Meier analysis allows two kinds of survival data: the event data and the censored data. We classified it as ‘event data’ when the larva died, and classified it as ‘censored data’ when the larva pupated or remained alive at the end of the experiment. The censored data allowed us to use the survival information of larvae that pupated or did not die during the experiment, so this replicate only affected the proportions of surviving to that date but did not add a mortality event. For the analyses of oxygen consumption, the effects of vial conditions (submerged, closed vial, and open vial), temperature (15°, 25°, or 35 °C), the medium where O_2_ was taken (air or water), and stage (larva or pupa) in O_2_ consumption, one or two-ways ANOVAs were performed, after verification of a normal distribution of datasets. A posteriori comparisons of significant ANOVAs were performed by means of the Tukey test. The significance threshold was chosen at 0.05 for all analyses.

## 3. Results

### 3.1. Survival Experiments

**Figure 1:**
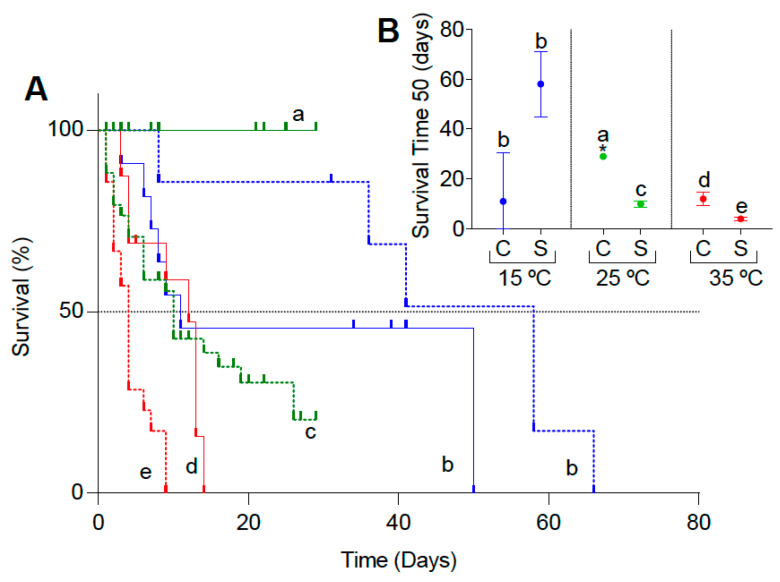
(**A**) Survival of *Ae. aegypti* larvae. Control (solid line) and submerged treatments (dotted line) at three temperatures: 15°, 25°, and 35 °C (blue, green, and red, respectively). The vertical-colored symbols represent the censored data. (**B**) Survival time 50 (estimated and 95% confidence intervals) of *Ae. aegypti* larvae at the control and submerged treatments at three temperatures: 15°, 25°, and 35 °C (blue, green, and red, respectively). Different letters indicate significant differences between conditions (submerged and control) and between temperatures in the submerged condition (*p* < 0.05). * It was not possible to estimate the survival time 50 due to the lack of mortality in the control treatment at 25 °C, so the value shown is the maximum survival time registered.

The survival of *Ae. aegypti* larvae were significantly affected by the temperature and immersion conditions (*p* < 0.05). In the control treatment (i.e., with access to air) at 25 °C, no death was registered, and the survival curve was significantly higher than the curve of the submerged treatment at the same temperature. In particular, at 25 °C, we ended the experiment on day 29 because only one larva from the submerged treatment was still alive. At 35 °C, the survival curve of the submerged larvae presented the lowest values with a higher negative slope, differing significantly from its control at the same temperature. Surprisingly, at 15 °C, the submerged treatment did not differ from the control (Figure 1A). The 50% mortality of submerged larvae differed with the temperature, being 58, 10, and 5 days at 15°, 25°, and 35 °C, respectively (Figure 1B). Remarkably, some individuals remained alive for as long as 30 days at 25 °C and 68 days at 15 °C. Finally, whereas we registered molts to pupae in control larvae, individuals kept submerged never molted. However, the percentage of pupation observed was relatively low, with a maximum at 25 °C (52.38%), followed by 35° (18.75%) and 15 °C (9.09%, Table 1) (Table 1).

### 3.2. Oxygen Consumption

#### 3.2.1. Larvae and Pupae at 25 °C

**Figure 2:**
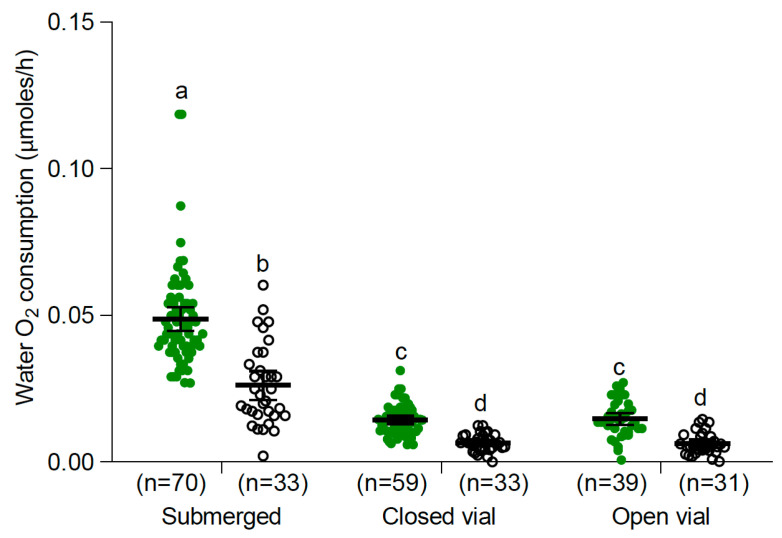
Consumption of water-dissolved O_2_ (mean and 95% confidence intervals) by *Ae. aegypti* larvae (green circles) and pupae (white circles) under three conditions: submerged, closed vial, and open vial at 25 °C. Different letters indicate significant differences between treatments (two-way ANOVA, F: 124.14, DF: 5; 259, *p* < 0.0001, Tukey multiple comparisons).

In all three conditions (submerged, closed vial, and open vial), *Ae aegypti* larvae and pupae evinced to consume measurable amounts of oxygen from the water (Figure 2). A significant difference in O_2_ consumption from the water was observed between the interaction of immature stages and the vial conditions (two-way ANOVA F: 124.14, DF: 5; 259, *p* < 0.0001). Larvae under the submerged condition presented the highest rate of water-dissolved O_2_ consumption, followed by pupae under the same vial conditions. In addition, larvae under closed vial and open vial conditions presented higher rates of O_2_ consumption than pupae under the same vial conditions, but their O_2_ consumption rates were always lower than larvae and pupa under submerged conditions (Figure 2). No significant difference was found between closed and open vial treatments for both immature stages. As the open vial condition did not provide additional information in the following measurements, we used the closed vial and the submerged conditions.

**Figure 3:**
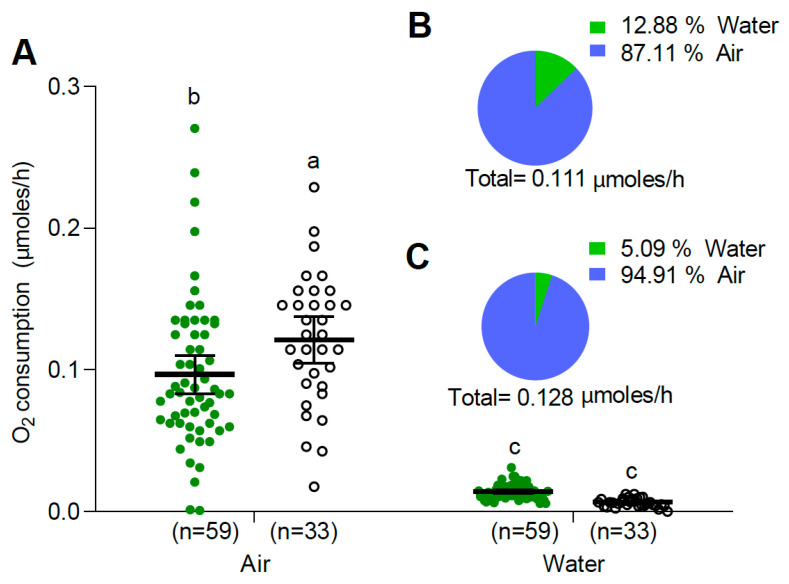
(**A**) O_2_ consumption (mean and 95% confidence intervals) obtained from air and water of *Ae. aegypti* larvae (green circles) and pupae (white circles) in the closed vial condition. Different letters indicate significant differences between treatments (two-way ANOVA, F: 109.72, DF: 3; 180, *p* < 0.0001, Tukey multiple comparisons). Percentage of oxygen consumption of *Ae. aegypti* larvae (**B**) and pupae (**C**), obtained from air and water.

**Figure 4:**
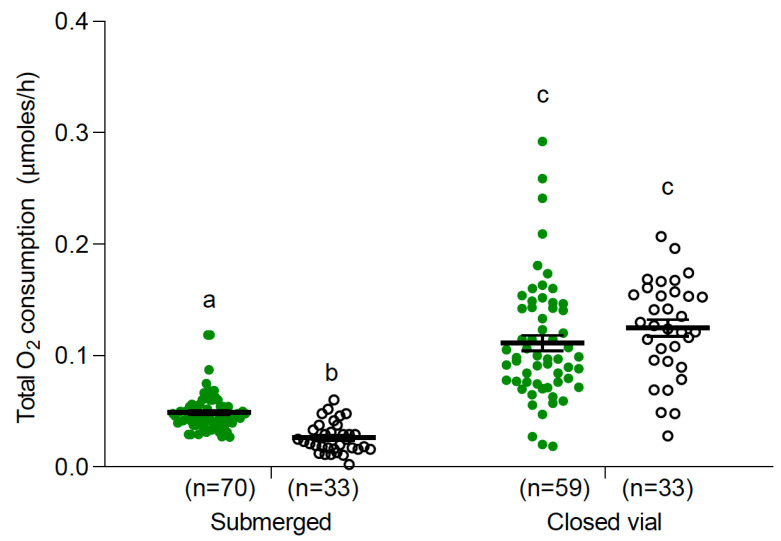
Total O_2_ consumption (mean and 95% confidence intervals) of *Ae. aegypti* larvae (green circles) and pupae (white circles) with (closed vial) and without (submerged) access to air at 25 °C. Different letters indicate significant differences between treatments (two-way ANOVA, F: 71.40, DF: 3; 191, *p* < 0.0001, Tukey multiple comparison).

Pupae presented higher oxygen consumption from the air than larvae under closed vial conditions (two-way ANOVA, F: 109.72, DF: 3; 180, *p* < 0.0001, Figure 3). The air O_2_ consumption of both immature stages tested was significantly higher than their water O_2_ consumption (*p* < 0.05, Figure 3). The dual rate of O_2_ consumption, adding air and water O_2_ consumption of closed vial treatment, were 0.111 and 0.128 µmoles/h for larvae and pupae, respectively. Larvae obtained most of their oxygen from the air but about 13% from water, while pupae obtained almost 95% from the air and the rest from water (Figure 3B,C). Finally, the dual (air + water) O_2_ consumption in closed vial condition was significantly higher than O_2_ consumption from the water of the submerged condition (two-way ANOVA, F: 68.85, DF: 3, *p* < 0.0001, Figure 4).

#### 3.2.2. Effect of Temperature on Oxygen Consumption (Q_10_ of Larvae)

The O_2_ consumption from the air of *Ae. aegypti* larvae in closed vial condition significantly varied with temperature (n = 12; one-way ANOVA, F: 33.46, DF: 2, *p* < 0.0001). The highest O_2_ consumption was observed at 35 °C, followed by 25 °C, and the lowest at 15 °C. The calculated Q_10_, which indicates the temperature sensitivity of oxygen consumption, was 1.47 between 15° and 25 °C, and 1.66 between 25° and 35 °C, so the overall mean Q_10_ across the experiment was 1.56. Also, the consumption of water-dissolver O_2_ of *Ae. aegypti* larvae when they were submerged significantly varied with temperature (n = 24; one-way ANOVA, F: 18.09, DF: 2, *p* < 0.0001). The highest O_2_ consumption was observed at 35 °C, followed by 25 °C, and the lowest at 15 °C. The calculated Q_10_ between 15° and 25 °C was 2.21, and between 25° and 35 °C was 1.45, so the overall mean Q_10_ across the experimental temperatures was 1.83.

#### 3.2.3. *Aedes albopictus*

**Figure 5:**
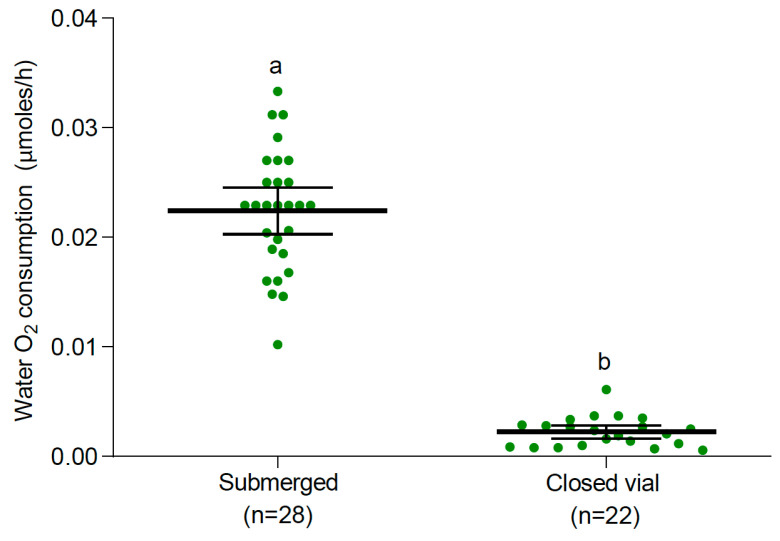
Consumption of O_2_ from water (mean and 95% confidence intervals) by *Ae. albopictus* larvae under two conditions: submerged and closed vial. Different letters indicate significant differences between treatments (one-way ANOVA, F: 278.36, DF: 1, *p* < 0.0001).

**Figure 6:**
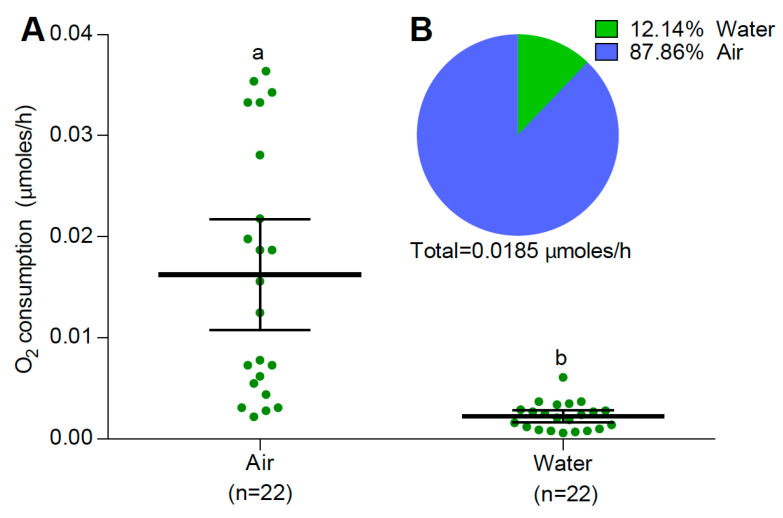
(**A**) O_2_ consumption (mean and 95% confidence intervals) obtained from air and water of *Ae. albopictus* larvae in the closed vial condition. (**B**) Percentage of oxygen consumption of *Ae. albopictus* larvae obtained from air and water. Different letters indicate significant differences between treatments (one-way ANOVA, F: 27.97, DF: 1, *p* < 0.0001).

**Figure 7:**
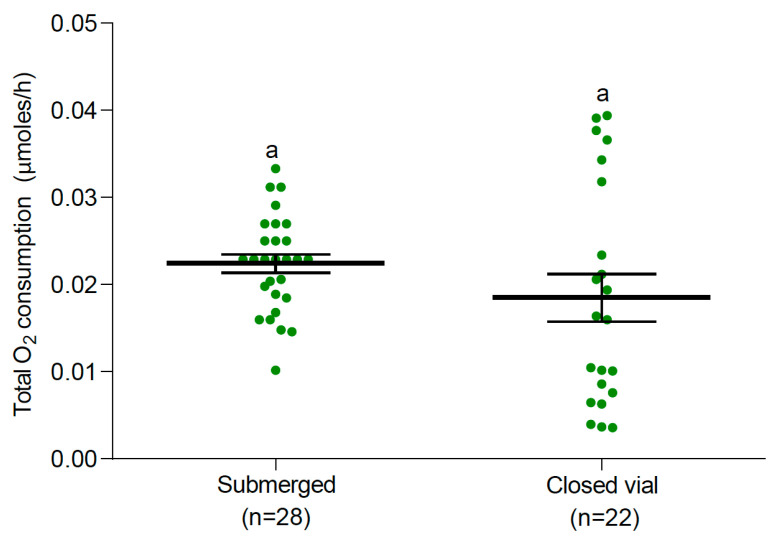
Total O_2_ consumption (mean and 95% confidence intervals) of *Ae. albopictus* larvae with (closed vial) and without access to air (submerged). Similar letters indicate non-significant differences between treatments (one-way ANOVA, F: 0.99, DF: 1, *p* = 0.325).

Similar patterns of O_2_ consumption were observed in *Ae. albopictus* at 25 °C. A significant difference in O_2_ consumption from the water was observed between vial conditions (one-way ANOVA, F: 278.36, DF: 1, *p* < 0.0001, Figure 5). *Ae. albopictus* larvae from the submerged condition treatment presented significantly higher O_2_ consumption than larvae from the closed vial (Figure 5). In the closed vial condition, the mean dual (air + water) rate of O_2_ consumption was 0.0185 μmoles of O_2_/h per larvae. They consumed a significantly higher amount of O_2_ (around 88%) from the air than from water (one-way ANOVA, F: 27.97, DF: 1, *p* < 0.0001, Figure 6). Interestingly, no significant differences were observed in the total O_2_ consumption of *Ae. albopictus* larvae between water-dissolved O_2_ consumption and dual O_2_ consumption (when they had access to both water and air) in the closed vial condition (one-way ANOVA, F: 0.99, DF: 1, *p* = 0.325, Figure 7). Conversely, temperature had a notable impact on the rate of O_2_ consumption from water (one-way ANOVA, F: 77.52, DF: 2, *p* < 0.0001). Specifically, the rate of O_2_ consumption did not show a significant difference between 15 °C (n = 20) and 25 °C (n = 28). However, a significant increase was observed when comparing either 15 °C or 25 °C to 35 °C (n = 20). The Q_10_, representing the temperature sensitivity of O_2_ consumption, was calculated to be 1.47 between 15 °C and 25 °C and 3.01 between 25 °C and 35 °C. Consequently, the average Q_10_ across the temperature range of 15 °C to 35 °C was determined to be 2.24.

## 4. Discussion

We report the first simultaneous quantitative data on oxygen consumption, from both air and water, by larvae and pupae of two major disease vectors, *Aedes aegypti* and *Ae. albopictus*. In both cases, not surprisingly, most of the oxygen consumed comes from the air, but not all. The proportion obtained from water is small but physiologically significant, just enough to survive, and much smaller in pupae, which obtain practically all the oxygen they consume from the air. A possible explanation could be differences in gas permeability, given that pupae possess a double cuticle, its own and that of the pharate adult, reducing the capacity of gas exchange between the insect and the water [17]. It is interesting to note that with these differences in the source (i.e., atmospheric or water dissolved), the total oxygen consumption is similar in larvae and pupae. Even though pupae could be much less active than larvae, they are experiencing metabolically-demanding body remodeling during adult metamorphosis.

Oxygen dissolved in water can differ in concentration, depending on water temperature and the photosynthetic activity of aquatic plants. Oxygen-saturated water and low temperatures allow the larvae of some mosquito species to survive prolonged submersion. For example, the North American pitcher plant mosquito *Wyeomyia smithii* [18] and the treehole anopheline *Anopheles barberi* [19] overwinter predominantly as larvae under ice [20]. In addition, the fourth instar larvae of *Ae. flavescens* spend most of their time among vegetation at the bottom of pools [21]. *Anopheles stephensi* larvae, which feed at considerable depth, were much more tolerant of prolonged submersion than *An. sacharovi* and *An. atroparvus,* which feed mainly at the water surface [22]. Large, thin-walled, and densely tracheated cephalic and anal papillae also allow *Culiseta morsitans* [23] and *Ae. argenteopunctatus* [24] to survive prolonged submersion.

Although there have been reiterated reports of mosquito larvae tolerating long submersion periods, probably breathing underwater, the phenomenon has remained anecdotal, and its physiological and practical significance for suffocation control methods is barely considered [3,4,9]. In our experiments, when completely submerged, larvae were able to survive for days, weeks, or even months, depending on the temperature of the water.

In this study, we measured the atmospheric oxygen uptake by *Ae. aegypti* immatures at different developmental stages. The calculated mean oxygen uptake rates (with minimum and maximum values) were 2.33 mm^3^/h (ranging from 0.65 to 6.50 mm^3^/h) per larva and 2.91 mm^3^/h (ranging from 0.005 to 5.50 mm^3^/h) per pupa. Compared to the existing literature, our measured mean oxygen uptake rates were slightly lower, i.e., 4.84 mm^3^/h (ranging from 4.62 to 5.00 mm^3^/h) per larva and 3.61 mm^3^/h (ranging from 3.60 to 3.62 mm^3^/h) per pupa [17]. Despite differences in factors such as the number of immatures, methodological approaches to measuring oxygen concentrations, and the specific developmental stages assessed, our results are in close agreement with those reported in the literature, even though a circadian rhythm of O_2_ consumption has been observed with a 35% higher rate at the late evening compared with the lowest rate [25]. It should be mentioned that oxygen consumption in larvae and pupae of *Ae. aegypti* has been shown to experience up and down variation with time as well as stress factors, such as overcrowding, which may complicate direct comparison across different studies [26,27,28].

As expected for a poikilothermic and ectothermic organism, the water temperature has a significant effect on larval metabolism, reflected in the intensity of both aerial and aquatic respiration. This dependence is expressed quantitatively by the calculation of Q_10_ values for oxygen consumption and by the differential survival time of fully submerged larvae over a wide range of temperatures. Our Q_10_ values of 1.56 and 1.83 for *Ae. aegypti* larvae calculated between 15° and 35 °C from O_2_ consumption in water and air, respectively, were similar to the 1.9 calculated between 12° and 45 °C in pupae of the same species [29]. As expected, the lower the temperature, the longer the survival time observed. This fact can be explained by the modulation of the larval metabolism, reflected in a Q_10_ higher than 1, together with a higher O_2_ concentration in the water, since the solubility of oxygen increases as the temperature decreases. Therefore, in tropical areas or areas where human activities result in relatively warm temperatures, we could expect a lower capacity to tolerate prolonged immersion due to a lower oxygen availability.

Interestingly, the molting cycle was noticeably affected by the lack of access to atmospheric air. None of the larvae in the prolonged submersion experiments were able to molt normally. Oxygen obtained exclusively from the water proved to be sufficient for survival and swimming (submerged larvae did not remain immobile) but not for molting. Only larvae kept at the lower temperature showed some signs of incomplete ecdysis after many weeks under water, indicating that the first steps of the molting process had taken place. It is likely that ecdysis is too energetically costly to complete under these conditions [30]. Given that the measured acquisition rate of water-dissolved oxygen is relatively low, additional atmospheric air seems to be required for successful pupation. If this were the case for *Ae. albopictus*, where oxygen consumption does not increase upon contact with atmospheric air, one would expect them to be able to pupate successfully even when completely submerged. Another possible limitation could be the requirement for a ‘significant’ amount of atmospheric air to facilitate successful pupation [22]. Future survival experiments with submerged *Ae. albopictus* larvae may help to decide between these two ideas.

According to our results (Figure 4) and those of Fraenkel and Herford [16], fully submerged *Ae. aegypti* larvae would consume half as much oxygen as larvae swimming normally. However, this is not the case for *Ae. albopictus*, whose larvae seem to collect similar amounts of oxygen when fully submerged or attached to the surface (Figure 7), as they were able to compensate for the lack of atmospheric oxygen, increasing their efficacy in gathering water-dissolved oxygen. Long-term survival of 54 days has been previously reported for *Ae. aegypti* larvae kept at 30 °C, even when they were unable to access the water surface [22]. In addition, no successful pupation in larvae exposed to the same treatment was reported [4]. These results are consistent with our findings and indicate that not only can *Ae. aegypti* larvae obtain oxygen directly from water, but that submerged larvae are unable to continue their development into pupae. Additionally, the observed failure to pupate could also be related to the need for the mosquito to obtain some air for separating the cuticle of the last-instar larva from the cuticle of the pupa and to release the trumpets that attach the pupa to the surface [10].

In a context with larval control by suffocation, Hagstrum [31] measured the aerial and aquatic respiration of larvae of several species in the presence of petroleum oils in the water. His study suggested that for *Ae. aegypti* aquatic respiration may account for approx. 5–20% of aerial respiration, rather than 50% as previously suggested by Fraenkel and Herford [16]. This agrees with our results where aquatic respiration of *Ae. aegypti* larvae was about 13%. Hagstrum [31] also reported delayed mortality in *Ae. aegypti* larvae, despite their tracheae being blocked with petroleum oil, and noted that these larvae were unable to pupate.

## 5. Conclusions

Our study sheds light on the respiratory physiology of the aquatic stages of mosquitoes by analyzing the survival and oxygen uptake of mosquito larvae and pupae with and without access to air and measuring the effect of temperature on these variables. For the first time, we provide quantitative data on oxygen consumption from air and water, measured separately and simultaneously. Using state-of-the-art respirometry, we show that the consumption of water-dissolved oxygen by mosquito larvae is not negligible, challenging the idea that they use only atmospheric oxygen.

## Figures and Tables

**Table 1:** Percentage of pupation (sample number) of *Ae. aegypti* larvae at control and submerged treatments at three temperatures: 15°, 25°, and 35 °C.

Temperature (°C)	Control	Submerged
15°	9.09% (n = 11)	0 (n = 7)
25°	52.38% (n = 21)	0 (n = 36)
35°	18.75% (n = 16)	0 (n = 21)

## Data Availability

The data presented in this study are available in Supplementary Material here.

## Supplementary Materials

The following supporting information can be downloaded at https://www.mdpi.com/article/10.3390/insects15020099/s1: Figure S1: Setup for testing larval survival at different temperatures; Tables S1 and S2: Complete dataset on supplementary Excel file.
