# Supplementary material for: Challenging Popular Belief, Mosquito Larvae Breathe Underwater
Source: Insects. 2024 Feb 1;15(2):99. doi: 10.3390/insects15020099 (PMC10889830; doi:10.3390/insects15020099)
Supplement: Supplementary file 1 [file insects-15-00099-s001.zip › Supplementary material.pdf]

Supplementary material

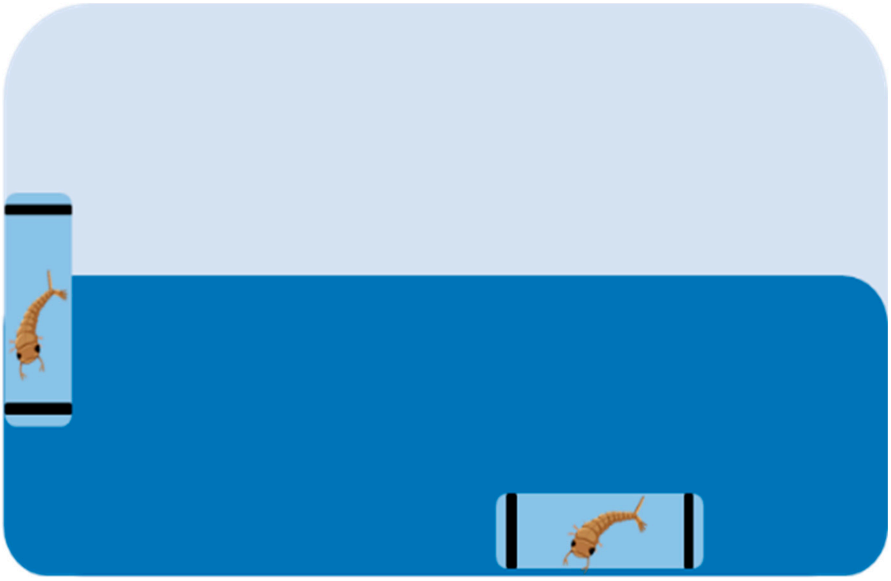

Figure S1. Setup for testing larval survival at different temperatures.

Table S1. Species, stages, vial conditions and medium (water and air) where the oxygen consumption was measured at 25°C.

	Aedes aegypti		*Aedes albopictus*
	Larvae	Pupae	Larvae
Submerged condition	Water	Water	Water
Closed vial condition	Water + Air	Water + Air	Water + Air
Open vial condition	Water	Water	---

Table S2. Oxygen consumption measurements for Q_{10} calculations at 15°, 25° and 35°C.

	***Aedes aegypti* larvae**	***Aedes albopictus* larvae**
Submerged condition	Water	Water
Closed vial condition	Air	---
